# Low catalytic activity is insufficient to induce disease pathology in triosephosphate isomerase deficiency

**DOI:** 10.1002/jimd.12105

**Published:** 2019-06-11

**Authors:** Joanna Segal, Michael Mülleder, Antje Krüger, Thure Adler, Manuela Scholze‐Wittler, Lore Becker, Julia Calzada‐Wack, Lillian Garrett, Sabine M. Hölter, Birgit Rathkolb, Jan Rozman, Ildiko Racz, Ralf Fischer, Dirk H. Busch, Frauke Neff, Martin Klingenspor, Thomas Klopstock, Nana‐Maria Grüning, Steve Michel, Beata Lukaszewska‐McGreal, Ingo Voigt, Ludger Hartmann, Bernd Timmermann, Hans Lehrach, Eckhard Wolf, Wolfgang Wurst, Valérie Gailus‐Durner, Helmut Fuchs, Martin H. de Angelis, Heinrich Schrewe, Mariia Yuneva, Markus Ralser

**Affiliations:** ^1^ The Molecular Biology of Metabolism Laboratory, Francis Crick Institute London UK; ^2^ Max Planck Institute for Molecular Genetics Berlin Germany; ^3^ German Mouse Clinic, Institute of Experimental Genetics, Helmholtz Zentrum München German Research Center for Environmental Health (GmbH) Neuherberg/Munich Germany; ^4^ Institute for Medical Microbiology, Immunology, and Hygiene Munich Germany; ^5^ Friedrich‐Baur‐Institute, Department of Neurology Ludwig‐Maximilians‐Universität München Munich Germany; ^6^ Institute of Pathology, Helmholtz Zentrum München German Research Center for Environmental Health (GmbH) Neuherberg/Munich Germany; ^7^ Institute of Developmental Genetics, Helmholtz Zentrum München German Research Center for Environmental Health (GmbH) Neuherberg/Munich Germany; ^8^ Chair for Molecular Animal Breeding and Biotechnology, Gene Center Ludwig‐Maximilians‐Universität München Munich Germany; ^9^ Member of German Center for Diabetes Research (DZD) Neuherberg/Munich Germany; ^10^ Molecular Nutritional Medicine Else Kröner‐Fresenius Center, TUM Freising‐Weihenstephan Germany; ^11^ ZIEL – Institute for Food and Health Technical University Munich Freising‐Weihenstephan Germany; ^12^ Munich Cluster for Systems Neurology (SyNergy) Adolf‐Butenandt‐Institut, Ludwig‐Maximilians‐Universität München Munich Germany; ^13^ Deutsches Zentrum für Neurodegenerative Erkrankungen e. V. (DZNE) Site Munich Munich Germany; ^14^ Chair of Developmental Genetics TUM Freising‐Weihenstephan Germany; ^15^ Chair of Experimental Genetics Center of Life and Food Sciences Weihenstephan, TUM Freising‐Weihenstephan Germany; ^16^ Cambridge Systems Biology Centre and Department of Biochemistry University of Cambridge Cambridge UK; ^17^ Department of Biochemistry Charitè Universitätsmedizin Berlin Berlin Germany; ^18^ Oncogenes and Tumour Metabolism Laboratory The Francis Crick Institute London UK

**Keywords:** active site mutation, glycolytic enzymopathy, hemolytic anemia, protein stability disorder, site‐directed mutagenesis, triosephosphate isomerase deficiency

## Abstract

Triosephosphate isomerase (TPI) deficiency is a fatal genetic disorder characterized by hemolytic anemia and neurological dysfunction. Although the enzyme defect in TPI was discovered in the 1960s, the exact etiology of the disease is still debated. Some aspects indicate the disease could be caused by insufficient enzyme activity, whereas other observations indicate it could be a protein misfolding disease with tissue‐specific differences in TPI activity. We generated a mouse model in which exchange of a conserved catalytic amino acid residue (isoleucine to valine, Ile170Val) reduces TPI specific activity without affecting the stability of the protein dimer. TPI^Ile170Val/Ile170Val^ mice exhibit an approximately 85% reduction in TPI activity consistently across all examined tissues, which is a stronger average, but more consistent, activity decline than observed in patients or symptomatic mouse models that carry structural defect mutant alleles. While monitoring protein expression levels revealed no evidence for protein instability, metabolite quantification indicated that glycolysis is affected by the active site mutation. TPI^Ile170Val/Ile170Val^ mice develop normally and show none of the disease symptoms associated with TPI deficiency. Therefore, without the stability defect that affects TPI activity in a tissue‐specific manner, a strong decline in TPI catalytic activity is not sufficient to explain the pathological onset of TPI deficiency.

## Abstract 2

A new mouse model shows that reducing enzyme activity, without affecting the overall structure of triosephopshate isomerase (TPI), does not induce TPI deficiency‐like symptoms in mice.

## 1 INTRODUCTION

Triosephosphate isomerase (TPI) deficiency was among the first human genetic disorders discovered by systematic enzymological screens more than 60 years ago.^1^ The syndrome is characterized by severe progressive neuromuscular degeneration (often manifesting as early as the first 7 months of life), neurologic dysfunction that is associated with impaired synaptic vesicle dynamics, hemolytic anemia and associated susceptibility to infections, and episodic hypotonia.^2^, ^3^, ^4^ Typically, patients are severely and systemically affected and do not reach the age of five. A noticeable exception are two brothers of Hungarian origin who have a milder form of the disease (caused by a unique compound‐heterozygous genotype).^2^

TPI deficiency is recessively inherited and caused by pathogenic genetic variants that alter the coding sequence of the *TPI1* gene on chromosome 12q19.^4^, ^5^ The native TPI enzyme is present as a 2× 27 kDa homodimer and fulfills an essential metabolic function by catalyzing the interconversion of the three carbon sugar phosphates, glyceraldehyde 3‐phosphate (Ga3P) and dihydroxyacetone phosphate (DHAP), in glycolysis.^6^ A substantial reduction of enzymatic activity is reported in all TPI deficient patients, as well as an accumulation of the TPI substrate, DHAP, indicating that glycolytic flux is affected.^4^ Reduced glycolytic flux and consequent energy disturbance, alterations in lipid metabolism, and the interconversion of DHAP to toxic methylglyoxal and consequent increase in oxidative stress have been discussed as biochemical causes for, or at least contributors to, TPI deficiency.^4^, ^7^, ^8^ Such features would be consistent with a typical metabolic enzymopathy, defined as the biochemical consequences of reduced enzymatic activity.

However, other observations are in conflict with the typical enzymopathy hypothesis. First, TPI is considered the “least rate limiting” enzyme of glycolysis, with only a fraction of its in vitro activity required to enable sufficient glycolytic flux to sustain normal cellular function.^9^ Hence, it is not clear if the partial loss of TPI enzyme activity would phenotypically penetrate and explain the disease symptoms. In parallel, it was noted early on that the population frequency of heterozygous TPI deficient alleles is higher than the actual disease prevalence would predict.^10^, ^11^, ^12^, ^13^, ^14^ The literature proposed two hypotheses to explain this discrepancy. The first suggests an evolutionary advantage of being a heterozygous carrier, which would maintain a higher frequency of heterozygote carriers in a population.^15^ The second possible explanation is that only a subset of the alleles that reduce TPI's enzyme activity induce disease, while other allelic combinations, although reducing the activity to a similar extent, would not result in a clinical disease. Thus far, a heterozygote advantage of TPI deficient alleles could not be substantiated. For instance, in a large survey of centenarians, TPI deficient alleles were neither enriched nor diminished.^16^ Instead, the TPI deficient patients discovered to date are consistent with the alternative hypothesis that only some of the deficient alleles cause TPI deficiency. Indeed, while many mutations could hypothetically affect TPI's enzymatic activity, symptomatic penetrance in the homozygous state is only seen for a single allele, a G‐C transversion in codon 104, which causes a conserved residue exchange from glutamic acid to aspartic acid.^4^, ^5^, ^17^, ^18^, ^19^, ^20^

The Glu104Asp amino acid substitution is located in the dimer interface and does not directly influence the catalytic function of TPI; transgenic yeast models expressing the allele show catalytic functionality but revealed altered affinities of the TPI dimer subunits in vivo.^21^ A crystal structure revealed the reduced dimer stability caused by the Glu104Asp substitution is explained by the disruption of an elaborate conserved network of buried water molecules that bridge the two subunits.^20^

A few years ago our research revealed interesting features of another allele, TPI^Ile170Val^. The TPI^Ile170Val^ allele is associated with TPI deficiency only in a compound‐heterozygous context, that is, disease‐causing only in combination with the common pathological allele, TPI^Glu104Asp^, and hence, not of major clinical relevance.^17^ However, the TPI^Ile170Val^ allele results in a direct reduction in catalytic activity due to the exchange of a conserved catalytic residue. Purified TPI^Ile170Val^ shows approximately 5% activity compared to wild type TPI in vitro resulting from changes in the key enzyme parameters, with an altered affinity for the substrate.^22^ However, this variant leaves the TPI dimer interface and secondary structure intact. The purified TPI^Ile170Val^ enzyme is fully folded and shows similar stability to wild type TPI alleles in vitro, according to CD spectroscopy.^22^ Due to the difference between the TPI^Ile170Val^ allele and the common pathogenic allele, TPI^Glu104Asp^, in terms of catalytic activity and structural stability, the TPI^Ile170Val^ mutant allele allows for the design of experiments that can disentangle the consequences of protein stability from those of a catalytic defect.

When introduced into *Drosophila* and paired with a TPI null allele, the human TPI^Ile170Val^ causes behavioral dysfunction in response to thermal and mechanical stress, symptoms that have been related to TPI deficiency.^23^ However, in other *Drosophila* models, TPI deficiency‐like phenotypes have also been induced by catalytically functional alleles in which reduced TPI deficiency is associated with dimer interface mutations or reduced protein stability. Two *Drosophila* mutant lines discovered in parallel named *sugar kill* and *wasted away* 24, 25 carry the same recessive hypomorphic TPI allele: a methionine to threonine substitution at the dimer interface (residue 80). The allele affects the stability of the protein, resulting in reduced TPI activity and neurological symptoms reminiscent of the human disease. Roland et al obtained a surprising result upon expressing the *sugar kill* allele in combination with a catalytically inactive TPI mutant allele, TPI^Met11Lys^.^26^ This compound heterozygous mutant was rescued from the behavioral and longevity phenotypes observed in the *sugar kill* homozygotes without restoring the overall TPI activity. Hence, in this particular model, it is not TPI activity but the degradation of the protein that causes all symptoms. Because of these contrasting observations in the various *Drosophila* models—a similar set of phenotypes is observed dependent and independent of a reduction in TPI specific activity—the *Drosophila* models remained inconclusive about the main cause of TPI deficiency.

The answer to the disease etiology question is not provided by two mouse models either. One of these carries an aspartate to glycine substitution at codon 49, positioned in the dimer interface,^27^ and a second has a phenylalanine to serine substitution at amino acid 57,^28^ which affects the stability of the protein and leads to its degradation. Both mouse models recapitulate the key symptom of TPI deficiency, hemolytic anemia, but, as they also both combine a stability defect with a decline in TPI activity that penetrates in a tissue‐specific manner, they do not enable the distinguishing of the consequences of activity decline from structural defect.

We speculated that the mouse models are compatible with a third hypothesis for explaining the etiology of TPI deficiency. In both models, TPI enzymatic activity varied greatly between tissues, with lowest activities to be measured in tissues, like brain, that were the most affected in patients. Hence, it was possible that the syndrome is caused by tissue‐specific activity differences. The symptoms would be caused by a TPI activity that falls below a pathological threshold in some tissues, while viability is maintained by sufficient TPI activity in other tissues.

In order to address this problem, we created a complementary mouse model in which we introduced the TPI^Ile170Val^ allele by site‐directed mutagenesis, reducing enzyme activity but leaving the dimer interface and protein stability unaffected. The C57BL/6J‐based animals have a approximately 15% residual TPI activity consistently in all examined tissues, which is a much stronger average activity decline than measured in human patients or the previous animal models of TPI deficiency. Without the tissue‐specific differences in TPI activity, TPI^Ile170Val/Ile170Val^ animals develop normally and show no signs of hemolytic anemia, neurological dysfunction, no major immune deficiency, motor neuron symptoms nor signs of metabolic syndrome—the phenotypes associated with the human disorder. Hence, in the absence of the protein stability defect that produces tissue‐specific TPI activity differences, a decline in TPI activity is not sufficient to cause symptoms of TPI deficiency.

## 2 RESULTS

### 2.1 TPI^Ile170Val/Ile170Val^ mice show a strong reduction in TPI activity uniformly across all examined tissues

**Figure 1:**
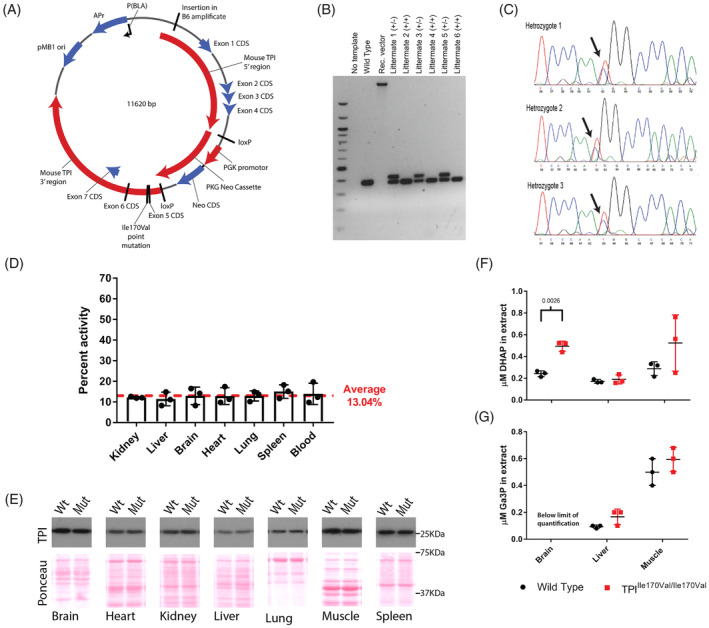
TPI^Ile170Val/Ile170Val^ mutation causes a reduction in enzymatic activity and affects glycolysis in vivo. A, Schematic diagram of the *Escheria coli* vector used for the generation of TPI deficient mouse, to introduce a mutation of residue 170 located in exon 5 of the *Tpi1* locus. B, Example genotyping of heterozygous (lane 4, 6, 8 “+/−”) and wild‐type (lane 5, 7, 9, “+/+”) littermates. C, Example sanger sequencing traces of the TPI locus of three heterozygous littermates. D, TPI activity of tissue lysates of TPI^Ile170Val/Ile170Val^ animals shown relative to wild‐type activity. Data are represented as mean ± SD of three independent experiments. E, Western blot to assess TPI protein levels in lysates from wild type and TPI^Ile170Val/Ile170Val^ animals. Blots show representative example of three biological repeats. Membranes stained with Ponceau‐S stain to show protein loading (F and G). Relative concentrations of the TPI substrates, DHAP and Ga3P, in extracts from wild type and TPI^Ile170Val/Ile170Val^ tissues determined by LC–MS/MS. Bars represent mean ± SD. *P* values shown only for significant differences (*P* < .05) between genotypes, calculated by Student's *t* test

In order to introduce the *TPI* ^*Ile170Val*^ mutation to mice, we cloned the mouse *TPI1* locus into an *Escheria coli* vector (pTREtight2, Addgene #19407), introduced the ATT to GTT transition by PCR mutagenesis and inserted a loxP‐flanked neoR cassette into the *Nhe*I site into *TPI1* intron 5 (Figure 1A). The vector was then used to generate TPI^Ile170Val/Ile170Val^ mice (Methods section, Figure 1B,C for an exemplary genotyping of an F1 litter). The mice were backcrossed for nine generations to C57BL/6J wild type animals.

We measured the TPI activity of the TPI^Ile170Val/Ile170Val^ mouse. While total TPI activities differ greatly between tissues, the TPI^Ile170Val/Ile170Val^ allele penetrates similarly across tissues in relative terms. In kidney, liver, brain, heart, lung, spleen, and blood, TPI^Ile170Val/Ile170Val^ mice exhibited about approximately 13% of total TPI activity compared to wild‐type animals (Figure 1D). This differs to the dimer interface mutants that exhibit substantial tissue differences in the relative penetrance of the enzyme defect.^28^, ^29^ In yeast, a partial compensation of TPI activity correlates with an upregulation of the enzyme protein level.^22^ However, in mice, no significant upregulation of the protein level was observed (Figure 1E). Consequently, the moderate increase of total TPI activity when comparing in vitro and in vivo experiments might be explained by post‐translational or metabolic regulation of TPI activity.

### 2.2 Substrate accumulation in TPI^Ile170Val/Ile170Val^ mice

Using mass spectrometry, we determined the concentrations of the TPI substrates, DHAP and Ga3P, in lysates of brain, liver and skeletal muscle of wild type and TPI^Ile170Val/Ile170Val^ mice. Glucose metabolism was altered in the TPI^Ile170Val/Ile170Val^ mice: we detected an increased level of the TPI substrate, DHAP, most strikingly in brain tissue (Figure 1F,G). These findings are consistent with the human disease in which patients are found to have high blood levels of DHAP.^4^ Ga3P levels are also elevated in liver and skeletal muscle of TPI^Ile170Val/Ile170Val^ mice. (Ga3P levels in brain tissues of wild type and TPI^Ile170Val/Ile170Val^ mice were below the level of quantification.)

### 2.3 TPI^Ile170Val/Ile170Val^ mice show no phenotypes associated with TPI deficiency

TPI^Ile170Val/Ile170Val^ mice were analyzed in a phenotypic screen. Sixty animals (15 male and 15 female, both wild type C57BL/6J and TPI^Ile170Val/Ile170Val^), all born from heterozygous crosses within 2 weeks, underwent systematic phenotyping.^30^, ^31^, ^32^ The systematic study revealed that, despite their substantial reduction in TPI activity, the animals display none of the phenotypes associated with TPI deficiency (summary of full screen can be found in supp. table 1).

**Figure 2:**
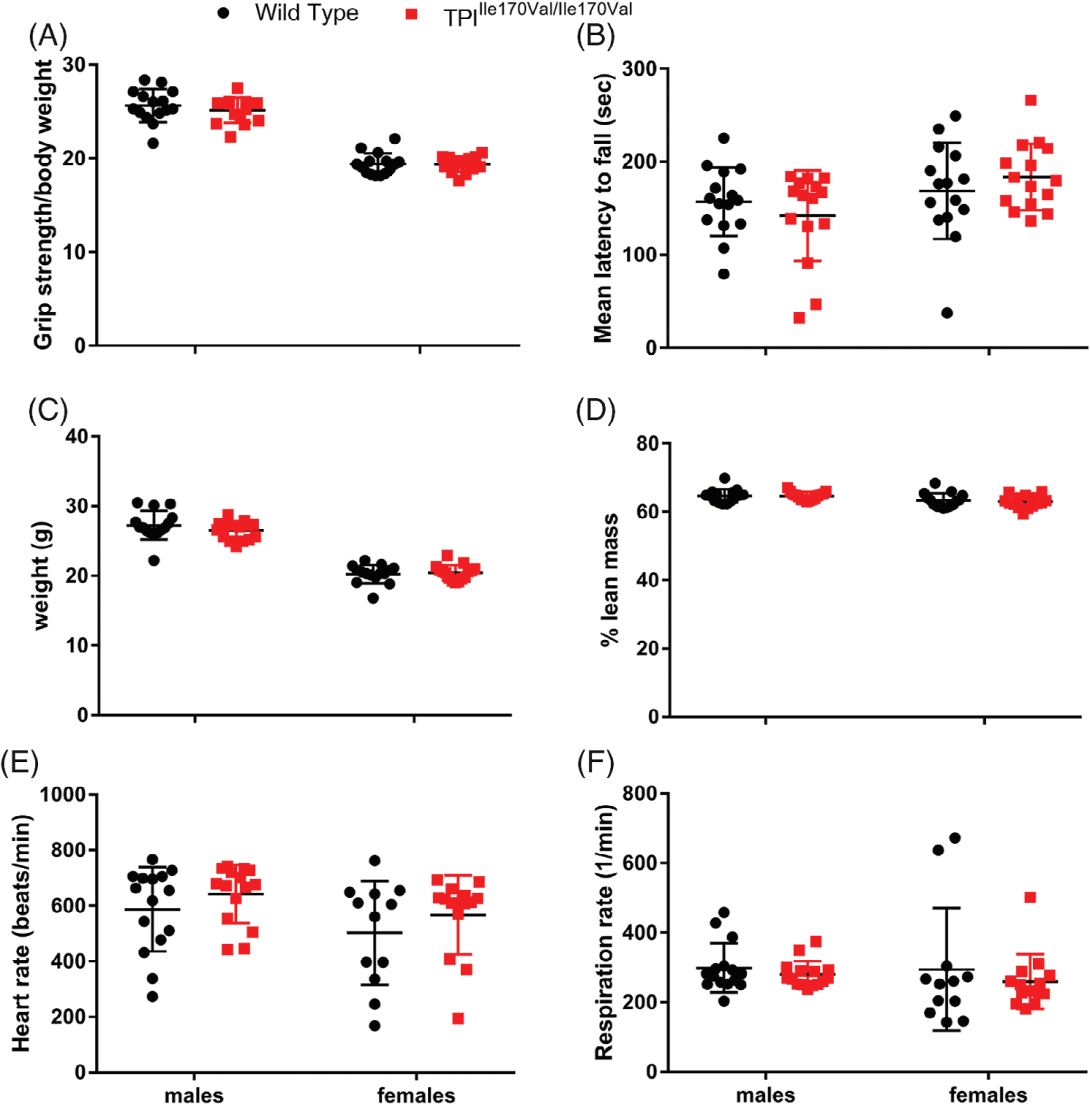
TPI^Ile170Val/Ile170Val^ mice show no signs of neurological, muscular, or cardiovascular alterations. Bars represent mean ± SD. *P* values shown only for significant differences (*P* < .05) between genotypes, calculated by Student's *t* test. A, Grip strength measured by a force meter on a mounted grid. Values shown relative to body weight. B, Balance measured using a rotarod. Mean latency to fall calculated over three trails. C, Body weight of wild type and TPI^Ile170Val/Ile170Val^ mice. D, Percentage lean mass calculated by qNMR. (E and F) Heart rate and respiration rate measured by echocardiogram

First, since TPI deficiency in humans is associated with progressive neuromuscular impairment, we assessed muscle function by measuring grip strength (Figure 2A). No significant differences were detected. Testing motor coordination with an accelerating rotarod also did not give any hints towards a neurological phenotype (Figure 2B). Body composition analysis revealed no indication of muscle wasting (Figure 2C,D). Because of muscle weakness, TPI deficient patients present with breathing and heart problems. However, we observed no significant difference in heart rate and respiration rate in TPI^Ile170Val/Ile170Val^ male and female mice (Figure 2E,F).

**Figure 3:**
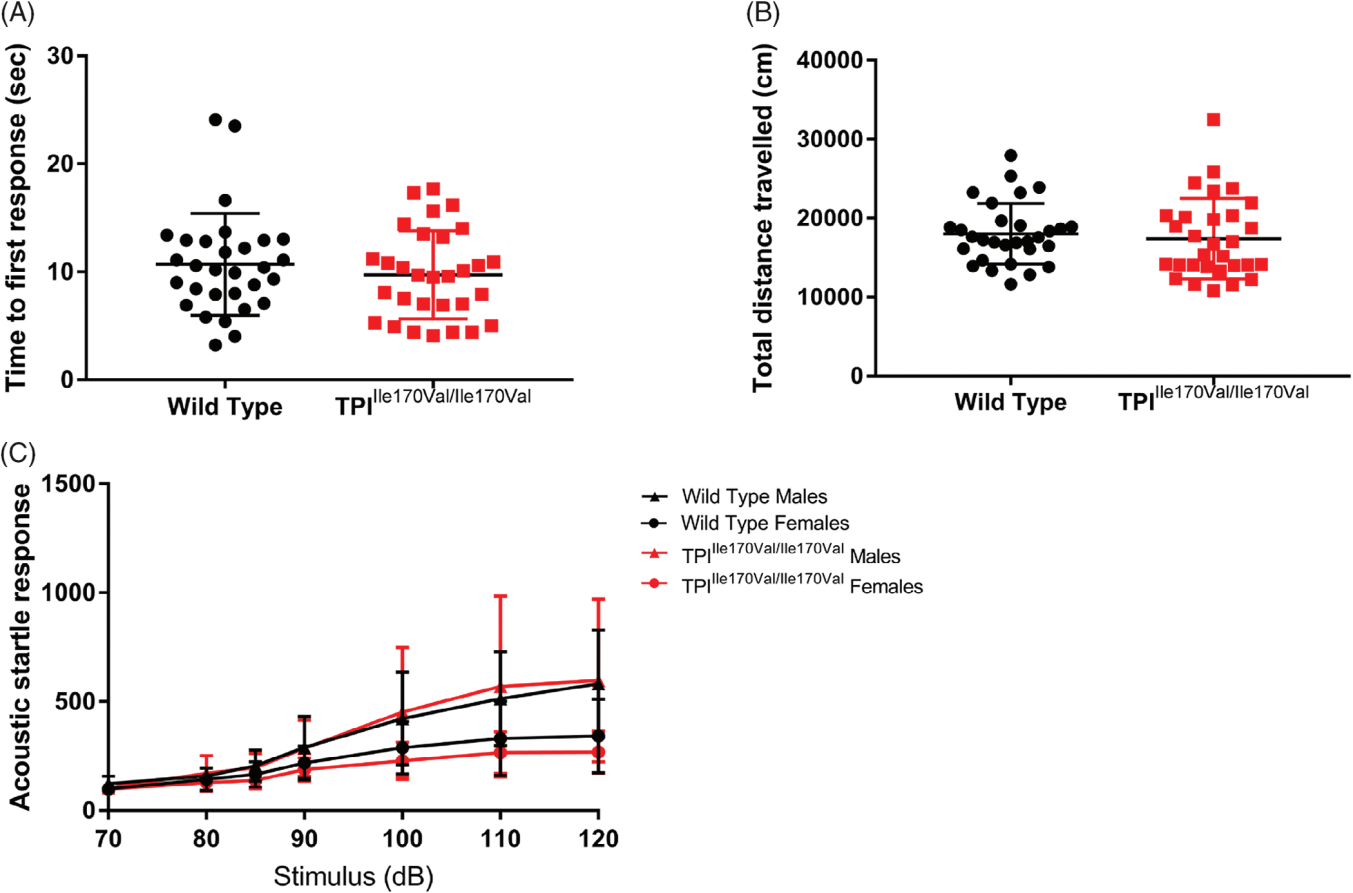
TPI^Ile170Val/Ile170Val^ mice display normal sensory and motor function. A and B, Bars represent mean ± SD. *P* values shown only for significant differences (*P* < .05) between genotypes, calculated by Student's *t* test. A, Mice tested on a hot plate and observed for the time taken to respond to heated surface of male and female mice pooled. B, Locomotor activity (total distance traveled) in the open field test of male and female mice pooled. C, Acoustic startle response measured over a series of increasing sound pressure stimulus volume. n = 15, mean ± SD

Some TPI deficient patients develop additional neurological symptoms, including intellectual disability. We challenged the TPI^Ile170Val/Ile170Val^ mice in behavioral tests sensitive to certain changes in central nervous system function (hot‐plate, open field, and prepulse inhibition) but found no differences of note (Figure 3).

**Figure 4:**
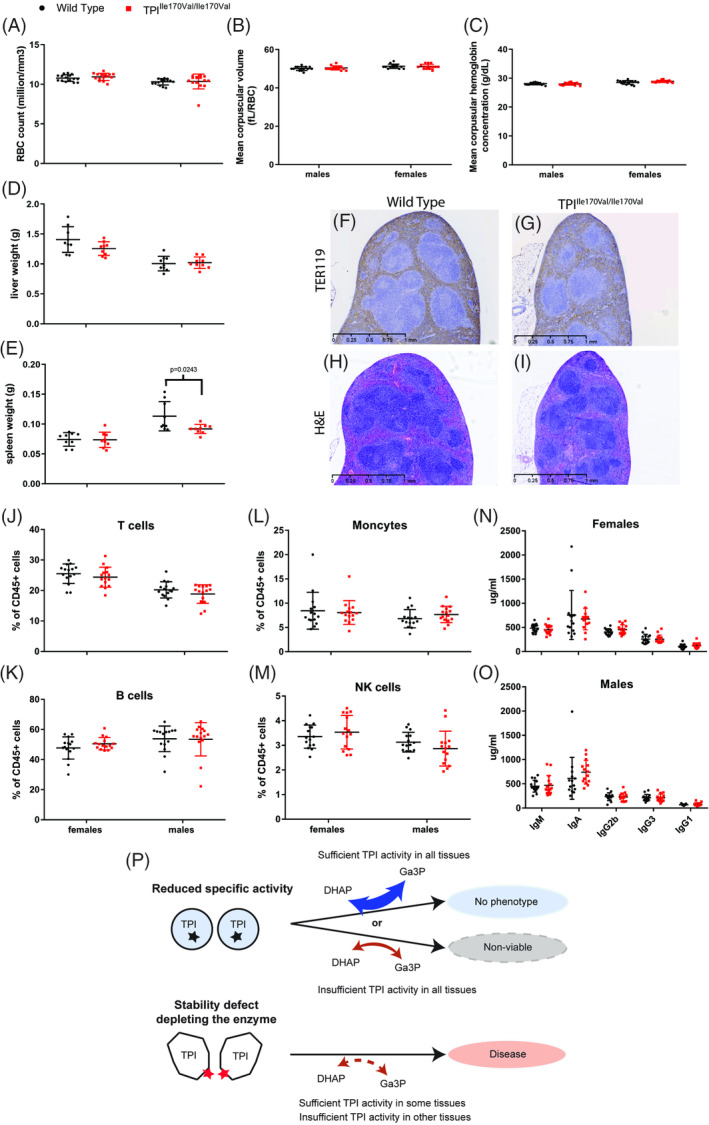
Clinical readouts of hemolytic anemia and associate immunodeficiency did not indicate pathology in TPI^Ile170Val/Ile170Val^ mice. A‐E, Bars represent mean ± SD. *P* values shown only for significant differences (*P* < .05) between genotypes, calculated by Student's *t* test. A‐C, Red blood cells, mean corpuscular volume and mean corpuscular hemoglobin calculated by abc‐Vet hematology analyzer. D and E, Liver and spleen weight. F and G, Representative images of spleens probed with TER119 antibody to stain the erythroid lineage. H and I, H&E staining of representative spleens. J‐M, Example cell populations in peripheral blood measured by flow cytometry and shown as a proportion of leukocytes (CD45+). Bars represent mean ± SD. *P* values shown for significant differences (*P* < .05) between genotypes, calculated by Student's *t* test. N and O, Immunoglobulin levels measured in plasma from male and female mice by multiplex analysis, P, Scheme: suggested model for the etiology of TPI deficiency

Similar results were obtained for hematology. Although hemolytic anemia is a main pathologic feature of TPI deficiency and the common characteristic of two mouse models that possess TPI alleles with structural defects,^27^, ^28^ there was no indication for such in the TPI active site mutant animals. In general, the data obtained did not provide evidence of any significant effect of the genotype on parameters measured (Figure 4A‐E). We examined the spleen and liver to detect any signs of hemolytic anemia, which is characterized by splenomegaly and reticulocytosis (increased production and circulation of reticulocytes), and often an enlarged liver. We observed a slight decrease in spleen size in female TPI^Ile170Val/Ile170Val^ mice, which is not consistent with hemolytic anemia (for which an increase in spleen size would is expected in most cases) and no difference in liver weight (Figure 4D,E). For better visualization of reticulocytes, we performed H&E staining and TER‐119 immunohistochemistry. The TER‐119 antibody reacts with cells of the erythroid lineage from the early erythroblast through mature erythrocyte stages in the red pulp of spleen.^33^ However, we detected no histological differences between mutant and control mice and observed no signs of increased reticulocyte production in the red pulp (Figure 4F‐I).

TPI deficient patients reportedly have a severely increased risk of infection. Therefore, we studied immunological parameters in TPI^Ile170Val/Ile170Val^ mice. The analysis of the proportions of leukocyte subsets in TPI mutant mice and littermate controls did not give evidence for a TPI‐related impaired leukocyte development (Figure 4J‐M). In addition, the measurement of basal immunoglobulin levels revealed similar basal immunoglobulin concentrations and a similar isotype distribution in mutants compared to controls (Figure 4N,O).

## 3 DISCUSSION

We have presented a mouse model that clarifies key missing aspects about the biochemical etiology of TPI deficiency, a severe, genetic metabolic disease known since the 1960s. The active site mutation of the TPI gene reduces the catalytic activity of the enzyme to approximately 13% of in vivo activity (Figure 1), which is a much more severe reduction in activity than observed in patients. Without affecting protein stability, the reduction in TPI activity was detected, in relative terms, uniformly across all examined tissues. This strong reduction in activity does not induce any disease symptoms associated with human TPI deficiency, including hemolytic anemia, neurological dysfunction, or shortened lifespan.^4^, ^21^, ^27^ As disease symptoms amplify in humans with age, we also investigated 2‐year old mice, but could not detect any notable phenotypes at this age either.

An important implication of our findings impacts drug development, which could focus on stabilizing the TPI enzyme structure and may not necessarily need to restore TPI enzymatic activity. Developing a drug that would replicate the complicated chemistry of the TPI enzymatic reaction is challenging by nature. Conversely, designing drugs that increase protein stability is an easier task. The latter can be achieved with molecules that show affinity to the protein, without necessarily needing to be metabolized or affect the highly optimal catalytic site of TPI.^34^, ^35^ We believe that aiming for the development of a TPI protein stabilizing agent would increase the chance of finding a suitable disease therapy.

Furthermore, our results suggest that in the diagnosis of TPI deficiency, enzyme activity measurements alone could be a poor predictor of disease penetrance. Nevertheless, we would like to stress that our results should not be misinterpreted as a claim that reduced TPI enzymatic activity, in itself, can be excluded as a contributing factor to the disease symptoms, nor that the sole cause of the disease symptoms must be a result of loss of an unknown second function or a new cellular problem caused by the TPI mutations. For instance, one study suggested that TPI deficiency could be caused by aggregation of the TPI protein.^7^ *Per se*, our mouse model does not contradict this hypothesis; however, it is also not confirmatory. Indeed, observations made with rare compound‐heterozygous cases imply that, although reduced TPI activity may not be sufficient to cause the disease symptoms, a reduced activity is required for penetrance: the rare compound‐heterozygous cases carry one allele with the enzyme dimerization defect (TPI^Glu104Asp^) and another allele that only reduces enzyme activity (eg, TPI^Ile170Val^).^17^ In these cases, the allele with reduced TPI activity is not able to compensate for the Glu104Asp allele, whereas, a wild‐type allele would. Conversely, when the Glu104Asp allele is heterozygous with a catalytically active pathological allele, the disease pathology does not penetrate to the same extent and results in a milder form of the disease.^2^ Such patterns of penetrance indeed point to a lack of TPI enzymatic activity to be the key disease causing agent.

These seemingly contradictory findings might be explained if one considers tissue specific factors. A previous mouse model of TPI deficiency, based on the dimer interface‐located residue mutation (methionine to threonine at residue 80), showed an average of approximately 35% loss of TPI activity. However, the activity ranged from approximately 55% in kidney to approximately 13% in brain.^29^ Similarly, a second mouse model of TPI deficiency, based on a mutation that also affects TPI protein stability, showed a range of activities in the tissues tested, from 48% in skeletal muscle to 10% in red blood cells.^28^ Conversely, the active site mutation of the TPI^Ile170Val^ allele penetrate—in relative terms—similarly across all tissues examined (Figure 1D). Hence, an intuitive explanation is that TPI^Ile170Val/Ile170Val^ mice are symptomless, as in each tissue a critical amount of TPI activity is reached, while in the (pathological) dimer interface mutants, in some tissues, TPI activity falls below the pathological threshold. Indeed, in the related metabolic disorder, ribose‐5‐phosphate isomerase (RPI) deficiency, tissue‐specific differences in the penetrance of the enzyme defect are considered to be the cause of the disease. Mutations that cause such tissue‐specific activity differences are far less likely to occur compared to null alleles, and, as a result, RPI deficiency is among the rarest genetic diseases known.^36^, ^37^ Thus, structural and metabolic defects can interact in a tissue‐specific manner, and a combination of both protein stability defects and lower catalytic activity may be required to explain the clinical outcome of these rare metabolic diseases, including TPI deficiency (Figure 4P).

## 4 MATERIALS AND METHODS

### 4.1 Generation of TPII^le170Val/Ile170Val^ mice

To create a vector for site‐directed mutagenesis of mouse *TPI1*, the wild type *TPI1* locus composed from C57BL/6J genomic DNA was PCR amplified and subcloned into an *E. coli* backbone (pTretight2, Addgene 19407). PCR mutagenesis was used to mutate codon 170 (ATT, encoding for isoleucine) in exon 5 to GTT, encoding for valine. Then, a loxP‐flanked *PGKneobpA* cassette was introduced into the *Nde*I site of TPI1 intron 4. The final vector was digested with *Sal*I and *Not*I to separate the mouse TPI locus from the vector backbone, and transformed into the 129×C57BL/6J hybrid embryonic stem cell line G4. One neomycin‐resistant cell line (2G5) was identified to have the integration on the *TPI1* locus and used to create a chimeric mouse. In brief, the cell line was expanded, injected into blastocysts and transplanted. Obtained chimera was crossed with Cre‐recombinase‐expressing C57BL/6J mouse line to remove the neomycin selection cassette. F1 littermates were tested for correct excision of the *NEO* ^*+*^ marker by PCR and re‐sequenced to verify that only the mutation in exon 5, and one loxP site in intron 4 was retained. This line was then backcrossed to the C57BL/6J mouse line for nine generations.

### 4.2 TPI activity assays

TPI activity was determined using a spectrophotometric assay as previously described.^29^, ^38^ Measurements were performed at 2‐3 different protein concentrations per tissue lysate and repeated independently three times with freshly dissected tissues from 20‐week‐old male mice. NADH loss was measured using a Tecan M1000 PRO plate reader. TPI activity was calculated at the point of maximal reaction rate and normalized to lysate‐free background activity.

### 4.3 Western blotting

Protein lysates were prepared from dissected tissues from 20‐week‐old male mice and western blots were performed using a polyclonal TPI serum (1:5000).^39^

### 4.4 Substrate measurements

Immediately following dissection, tissues from 20‐week‐old male mice were freeze clamped in liquid nitrogen and stored at −80°C. Tissues were ground to a fine powder over liquid nitrogen and extracted in a mix of chloroform, methanol, and water in a ratio of 40:40:20 with 0.1 M formic acid, as previously described.^40^

The samples were analyzed by tandem mass spectrometry on an Agilent 1290 liquid chromatography system coupled to an Agilent 6470 triple quadrupole mass spectrometer. The LC‐MS/MS method used was based on the Agilent Metabolomics dMRM Database and Method with a shortened chromatography. The compounds were resolved on a C18 column (Zorbax RRHD Extend‐C18, 2.1 × 100 mm, 1.8 μm; Agilent) with mobile buffer A1 (3% methanol, 10 mM tributylamine, 15 mM acetic acid), mobile buffer B1 (10 mM tributylamine, 15 mM acetic acid, 97% acetonitrile, 3% methanol), and mobile buffer B2 (acetonitrile) by gradient elution at a constant column temperature of 35°C. The gradient program started with 100% A and a flow rate of 0.35 mL/min. The organic fraction (B) was increased to 20% from 2 to 5 minutes and to 45% from 5 to 10 minutes. This was followed by a 2 minutes wash with 99% B1 and a 3 minutes wash of 99% B2 (1 mL/min) before returning to initial buffer conditions for equilibration at a flow rate of 0.6 mL/min for 1 minute and at a flow rate of 0.35 mL/min for 2 minutes, resulting in a total runtime of 18 minutes. The metabolites were quantified by external calibration (Sigma‐Aldrich 37442, G5251).

### 4.5 Phenotypic screen

A cohort of 60 mice were phenotypically analyzed at the German Mouse Clinic (GMC) in two standardized pipelines for systematic primary phenotyping as previously described,^30^, ^41^ with 15 mice per group utilized for each test (equal numbers of male and female, and mutant and wild‐type animals). All animals were age‐matched within 2 weeks of each other and ranged from 63 to 131 days old at the time of testing. All tests performed were approved by the responsible authority in the United Kingdom, as well as the district governments of Berlin and Upper Bavaria, Germany, respectively.

All institutional and national guidelines for the care and use of laboratory animals were approved by the responsible authority in the United Kingdom, as well as the district governments of Berlin and Upper Bavaria, Germany.

This manuscript does not contain studies with human subjects performed by any of the authors.

## CONFLICT OF INTEREST

None.

## AUTHOR CONTRIBUTIONS

Creation, design, and maintenance of the mouse model: J.S., A.K., M.S.‐W., N.‐M.G., S.M., B.L.‐M., I.V., L.H., H.S., M.R.

Metabolic measurements and Clin Chem.: M.M., B.T., B.R., E.W., J.R., M.K.

Conceptualization and writing of the paper V.G.‐D., H.F., M.H.d.A., H.L., M.Y., M.R.

Immunology experiments: T.A., D.H.B.

Neurology experiments: L.B., T.K.

Pathology experiments: J.C.‐W., F.N.

Behaviour experiments: L.G., S.M.H., W.W.

Nociception experiments: I.R.

Cardiovascular experiments: R.F.

## Supporting information


**Table S1.** Xxx.
