# Supplementary material for: Low catalytic activity is insufficient to induce disease pathology in triosephosphate isomerase deficiency
Source: J Inherit Metab Dis. 2019 Jun 11;42(5):839–49. doi: 10.1002/jimd.12105 (PMC7887927; doi:10.1002/jimd.12105)
Supplement: Supplementary file 1 — Table S1. Xxx. [file JIMD-42-839-s001.pdf]

Test	Description	Unit	Female TPI$^{Ile170Val/Ile170Val}$	Female wild type	Male TPI$^{Ile170Val/Ile170Val}$	Male wild type
**Acoustic_startle_PPI**	Percentage Prepulse inhibition - Global	%	48.6 (42.1, 57.7)	53.5 (45.8, 64.2)	54 (50, 60.2)	53.4 (44.7, 61.9)
**Acoustic_startle_PPI**	Percentage Prepulse inhibition - PP 67 dB;Percentage Prepulse inhibition - PP67	%	46.1 (38.9, 56.5)	49.2 (41.5, 64.1)	47.9 (30.8, 55.2)	55.3 (38.7, 56.9)
**Acoustic_startle_PPI**	Percentage Prepulse inhibition - PP 69 dB;Percentage Prepulse inhibition - PP69	%	47.7 (41.6, 55.9)	60.8 (44.1, 65.6)	54.5 (50.1, 64.2)	51.9 (46.8, 61.8)
**Acoustic_startle_PPI**	Percentage Prepulse inhibition - PP 73 dB;Percentage Prepulse inhibition - PP73	%	57.5 (40.9, 64.3)	58.7 (47.8, 62.7)	58.1 (54.6, 60.5)	56.2 (44.6, 65.1)
**Acoustic_startle_PPI**	Percentage Prepulse inhibition - PP 81 dB;Percentage Prepulse inhibition - PP81	%	57.8 (43.1, 64.3)	56.3 (46.1, 63.9)	60.2 (53.1, 64.9)	56.2 (51.2, 72.2)
**Acoustic_startle_PPI**	Acoustic Startle Response at 100 dB	dB	215.4 (162.9, 260.1)	259.6 (196.3, 353.6)	341.2 (280.2, 450.7)	409.6 (279, 542.5)
**Acoustic_startle_PPI**	Acoustic Startle Response at 110 dB	dB	289.2 (183.9, 340.6)	289.7 (245.6, 404.3)	423.8 (302.8, 618.7)	470.5 (372.5, 643.8)
**Acoustic_startle_PPI**	Acoustic Startle Response at 120 dB	dB	266.1 (183.6, 338.3)	291.1 (205.3, 406.6)	492.7 (307.1, 707.6)	629.8 (398, 759.8)
**Acoustic_startle_PPI**	Acoustic Startle Response at 70 dB	dB	95.8 (82.4, 109.5)	100.8 (79.8, 111.2)	101.5 (95.1, 120.1)	116 (108.9, 132.7)
**Acoustic_startle_PPI**	Acoustic Startle Response at 80 dB	dB	125 (95.8, 157.3)	124.9 (110.2, 155.4)	138.9 (136.2, 151.8)	154 (132.5, 179.8)
**Acoustic_startle_PPI**	Acoustic Startle Response at 85 dB	dB	127.5 (107.8, 154.6)	148.2 (123.6, 191.7)	170.9 (152.6, 234.3)	179.9 (153.1, 260.4)
**Acoustic_startle_PPI**	Acoustic Startle Response at 90 dB	dB	183.7 (150.7, 222.1)	202.7 (160.2, 259.4)	237.8 (210.2, 349.1)	251.2 (190.7, 336.6)
**Calorimetry**	Total food intake per single-housed mouse and day	g	2.8 (2.5, 3)	2.5 (2.2, 3.2)	2.8 (2.5, 3.3)	3.1 (2.7, 3.4)
**Calorimetry**	Body temperature	°C	36.4 (36, 36.6)	36.5 (35.9, 36.6)	36.1 (35.6, 36.4)	35.9 (35.8, 36.2)
**Calorimetry**	Water consumption in ml	ml	4.1 (3.8, 4.3)	4.5 (3.7, 4.6)	4.3 (4, 4.4)	3.9 (3.8, 4.3)
**Calorimetry**	Differential distance with reference to the sample interval	cm	960.2 (816.5, 1349.8)	1133.1 (932.1, 1332.1)	889.1 (792.4, 1039.9)	821.6 (687.7, 942.7)
**Calorimetry**	Mean heat production	kJ/h/animal	1.6 (1.6, 1.7)	1.7 (1.6, 1.7)	1.8 (1.8, 1.9)	1.8 (1.8, 1.9)

**Calorimetry**	Mean respiratory exchange rate	VCO2/VO2	0.9 (0.9, 0.9)	0.9 (0.9, 0.9)	0.9 (0.9, 0.9)	0.9 (0.9, 0.9)
**Calorimetry**	Mean carbon dioxide production	ml/(h animal)	72.3 (70.6, 74.6)	73.4 (69.3, 78.7)	80.6 (76.3, 83.7)	81.4 (77.3, 86.3)
**Calorimetry**	Mean oxygen consumption per time	ml/(h animal)	79.6 (77.2, 83.8)	81.3 (78.1, 83.9)	88.7 (84.9, 91.4)	89.5 (85.7, 93.5)
**Calorimetry**	Mean breaks X-beam, ambulatory movement: Breaks of any two different X-beams of light at the lower X level. Records beam-breaks of movements in which, for example light barrier Xn ist first interrupted followed by an interruption to a different light barrier Xm. The counter records the new interruption after a different light barrier has been interrupted first.	counts	602.8 (519.9, 837.9)	684.2 (565.6, 738)	552.8 (491.4, 670.8)	525.7 (418.8, 617.6)
**Calorimetry**	Mean breaks X-beam, fine movement: Two consecutive breaks of a single beam of light at the lower level X. The counter records the interruption of a light barrier that has been interrupted for the second time in succession without a different light barrier having first been interrupted.	counts	227.9 (218.9, 275.7)	244.2 (235.7, 261.9)	226.6 (210.9, 251.9)	213.6 (195.3, 246.1)
**Calorimetry**	Means breaks X-beam total (is equivalent to XA + XF). A light barrier interruption is counted as either an ambulatory or a fine movement.	counts	829.1 (740.2, 1116.1)	928.8 (806.6, 994.6)	779.4 (700.8, 925.7)	740.5 (616, 855.9)

**Calorimetry**	Mean break Y-beam, ambulatory movement: Breaks of any two different Y-beams of light at the lower Y level. Records beam-breaks of movements in which, for example light barrier Yn ist first interrupted followed by an interruption to a different light barrier Ym. The counter records the new interruption after a different light barrier has been interrupted first.	counts	569.1 (467.4, 722.3)	608.1 (516.2, 693)	524.5 (494.1, 646.9)	511.9 (393.5, 611.9)
**Calorimetry**	Mean breaks Y-beam, fine Movement: Two consecutive breaks of a single beam of light at the lower level Y. The counter records the interruption of a light barrier that has been interrupted for the second time in succession without a different light barrier having first been interrupted.	counts	231.9 (217.5, 268.4)	241 (231.1, 260.6)	228 (211.6, 251.4)	225.2 (194.4, 243)
**Calorimetry**	Mean breaks Z-beam, rearing movement: A break of a beam of light at the upper level Z. (No differentiation is made between ambulatory and fine movements.)	counts	132.5 (84.1, 156.1)	132 (93.2, 163.1)	95.2 (75, 131.1)	94 (82.8, 108.3)
**Clinical_chemistry**	Albumin concentration in plasma	g/l	28.8 (28.3, 29.4)	28.1 (27.7, 30.1)	27.4 (26.9, 27.9)	27.4 (26.9, 28.1)
**Clinical_chemistry**	alpha-amylase test which uses CNPG3 as substrate of the test reaction	U/l	564.54 (550.59, 605.48)	532.98 (493.2, 578.33)	692.6 (657.46, 728.56)	665.36 (640.94, 703.73)
**Clinical_chemistry**	alkaline phosphatase activity in plasma	U/l	154 (147, 166.5)	143 (138, 159)	96 (90.5, 101)	95 (87, 100.5)
**Clinical_chemistry**	Total bilirubin concentration in plasma	µmol/l	0.2 (0.2, 0.2)	0.2 (0.2, 0.2)	0.2 (0.2, 0.2)	0.2 (0.2, 0.2)
**Clinical_chemistry**	Calcium concentration in plasma	mmol/l	2.41 (2.39, 2.44)	2.46 (2.42, 2.48)	2.42 (2.38, 2.45)	2.41 (2.37, 2.43)
**Clinical_chemistry**	Cholesterol concentration in plasma	mmol/l	1.999 (1.917, 2.119)	2.087 (2.014, 2.194)	2.811 (2.572, 2.981)	2.777 (2.633, 2.925)
**Clinical_chemistry**	Chloride concentration in plasma	mmol/l	113.3 (111.9, 113.8)	114.2 (112.8, 115.1)	108.9 (108.2, 109.7)	108.4 (106.2, 110)

**Clinical_chemistry**	Creatinine concentration in plasma measured enzymatically	µmol/l	14.14 (13.97, 14.98)	14.76 (14.46, 15.91)	15.38 (15.07, 15.87)	14.85 (11.89, 16.13)
**Clinical_chemistry**	Iron concentration in plasma	µmol/l	25.2 (23.85, 30.9)	26.2 (21.6, 29.5)	19.9 (18.25, 21.6)	19.2 (18.3, 20.25)
**Clinical_chemistry**	Glucose concentration in plasma	mmol/l	11.53 (10.87, 12.8)	12.4 (11.21, 13.5)	11.82 (9.73, 12.25)	10.47 (9.54, 11.85)
**Clinical_chemistry**	Aspartate aminotransferase activity in plasma	U/l	50 (45.5, 68.5)	48 (45.5, 63)	51 (44.5, 63)	50 (43.5, 69.5)
**Clinical_chemistry**	Alanine aminotransefrase activity in plasma	U/l	27 (24, 32)	26 (22.5, 33)	34 (30, 36.5)	34 (26, 73.5)
**Clinical_chemistry**	Potassium concentration in plasma	mmol/l	4.3 (4, 4.4)	4 (3.8, 4.3)	4.4 (4.2, 4.5)	4.2 (4, 4.4)
**Clinical_chemistry**	Lactate concentration in plasma	mmol/l	11.32 (10.43, 11.92)	11.17 (10.24, 11.61)	11.72 (10.95, 12.02)	11.02 (10.6, 11.68)
**Clinical_chemistry**	Sodium concentration in plasma	mmol/l	151 (149, 151.5)	151 (150, 152)	148 (148, 149)	148 (146.5, 148)
**Clinical_chemistry**	Inorganic phosphate concentration in plasma	mmol/l	1.1 (1, 1.25)	1.3 (0.9, 1.45)	1 (0.9, 1.05)	1 (0.9, 1.15)
**Clinical_chemistry**	Total protein concentration in plasma	g/l	54.7 (52.2, 55)	54.1 (52, 56)	54.2 (53, 55)	54.2 (53, 55.6)
**Clinical_chemistry**	Triglyceride concentration in plasma	mmol/l	0.927 (0.839, 1.11)	0.798 (0.616, 0.837)	1.573 (1.438, 1.743)	1.659 (1.457, 1.965)
**Clinical_chemistry**	Unsaturated iron binding capacity in plasma	µmol/l	39.2 (28.6, 41.6)	37.8 (35.3, 39)	41.1 (39.1, 44.5)	43.6 (40.4, 45.7)
**Clinical_chemistry**	Urea concentration in plasma	mmol/l	9.57 (8.81, 10.56)	9.14 (8.39, 10.45)	10.44 (9.48, 11.03)	10.57 (9.81, 11.44)
**DEXA**	Body length (cm) from nose tip to base of the tail measured during narcosis	cm	9.5 (9.4, 9.7)	9.5 (9.4, 9.7)	9.7 (9.5, 9.9)	9.8 (9.5, 9.9)
**DEXA**	Fat mass of whole body scan excluding the skull	g	0.872 (0.16, 1.676)	0 (0, 0.99)	2.343 (0.661, 5.562)	3.909 (1.795, 5.997)
**DEXA**	Lean mass of whole body scan excluding the skull	g	17.093 (16.389, 17.795)	17.992 (16.889, 18.998)	20.559 (19.215, 22.216)	20.742 (19.437, 21.461)
**DEXA**	Soft mass of whole body scan excluding the skull	g	18.113 (17.675, 18.479)	17.925 (17.077, 18.312)	23.504 (22.05, 25.069)	23.868 (23.275, 26.428)
**DEXA**	Bone area of whole body scan excluding the skull	cm^2	4.21 (3.319, 6.668)	3.662 (2.789, 6.565)	4.628 (3.998, 9.854)	8.626 (4.845, 10.003)
**ECHO_VEVO2100**	Heart rate	bpm	618.07 (580.47, 635.08)	582.68 (381.63, 644.3)	676.06 (589.97, 723.64)	654.54 (493.73, 702.46)
**ECHO_VEVO2100**	Interventricular septum in diastole	mm	0.5 (0.43, 0.55)	0.53 (0.47, 0.57)	0.55 (0.51, 0.6)	0.52 (0.51, 0.57)
**ECHO_VEVO2100**	Interventricular septum in systole	mm	0.48 (0.45, 0.57)	0.55 (0.52, 0.61)	0.57 (0.53, 0.59)	0.55 (0.48, 0.56)
**ECHO_VEVO2100**	Left ventricular internal dimension in diastole	mm	2.32 (2.06, 2.69)	2.59 (2.49, 2.99)	2.48 (2.31, 2.73)	2.67 (2.36, 3.33)

**ECHO_VEVO2100**	Left ventricular internal dimension in systole	mm	1.36 (1.21, 1.57)	1.47 (1.21, 1.71)	1.38 (1.28, 1.61)	1.65 (1.37, 2.02)
**ECHO_VEVO2100**	Left ventricular posterior wall width in diastole	mm	0.5 (0.48, 0.58)	0.54 (0.46, 0.58)	0.53 (0.47, 0.56)	0.51 (0.5, 0.55)
**ECHO_VEVO2100**	Left ventricular posterior wall in systole	mm	0.5 (0.46, 0.54)	0.54 (0.49, 0.62)	0.5 (0.47, 0.54)	0.48 (0.45, 0.54)
**ECHO_VEVO2100**	Respiration Rate Echo	1/min	241.5 (224.83, 272.91)	256.75 (195, 280.49)	269.66 (252.19, 291.5)	282.35 (255.35, 300.05)
**Eye_size**	Axial length left eye	mm	3.533 (3.515, 3.55)	3.543 (3.513, 3.57)	3.598 (3.564, 3.607)	3.603 (3.554, 3.617)
**Eye_size**	Axial length right eye	mm	3.532 (3.492, 3.543)	3.563 (3.526, 3.578)	3.593 (3.571, 3.601)	3.611 (3.579, 3.628)
**Eye_size**	Body length	mm	97 (95, 98)	98 (96.5, 99)	102 (100.5, 103)	102 (100, 103)
**FACS_blood**	B-1 cells; CD19+CD5+	%	0.87 (0.76, 1)	0.96 (0.9, 1.16)	0.72 (0.68, 0.94)	0.81 (0.67, 0.91)
**FACS_blood**	B cells expressing high levels of IgD (as percentage of B cells)	%	88 (87, 89.2)	88.2 (87.7, 89.9)	89.5 (88.75, 90.75)	90.6 (89.85, 91.25)
**FACS_blood**	MHC II and B220 co-expressing B cells; CD19+MHC II+B220+	%	98 (97.65, 98.15)	98.1 (97.7, 98.45)	98.6 (98.1, 98.65)	98.8 (98.25, 99.1)
**FACS_blood**	B cells (CD19+ cells; percentage of living leukocytes)	%	49.3 (47.95, 51.6)	48.3 (46.2, 51.5)	55.5 (53.55, 59.5)	58.3 (50.3, 58.9)
**FACS_blood**	T cells (CD3+ cells; percentage of living leukocytes)	%	24 (22.05, 25.9)	26.7 (24.45, 27.4)	19.9 (16.95, 21.35)	20 (18.9, 21.2)
**FACS_blood**	CD4+ T cells (percentage of living leukocytes)	%	10.2 (9.29, 11.5)	11.6 (10.75, 12.45)	9.13 (7.8, 9.71)	9.59 (8.28, 10.25)
**FACS_blood**	CD25+ CD4+ T cells (regulatory T cells; percentage of CD4+ T cells)	%	4.2 (3.82, 4.78)	3.63 (3.18, 3.75)	4.9 (4.06, 5.05)	4.24 (3.4, 4.49)
**FACS_blood**	CD44 expressing CD4 T cells; CD4+CD3e+CD44+	%	2.59 (2.21, 3.09)	2.51 (2.04, 3.03)	2.24 (2.1, 3.66)	2.59 (2.13, 3.06)
**FACS_blood**	L-selectin expressing CD4 T cells; CD4+CD3e+CD62L+	%	69.1 (55.75, 73.85)	70.9 (60.65, 75.25)	73.9 (66.4, 77.6)	73.7 (70.6, 76.65)
**FACS_blood**	CD8+ T cells (percentage of living leukocytes)	%	11 (9.8, 11.55)	11.3 (10.65, 11.85)	8.16 (7.32, 9.18)	8.35 (7.59, 8.79)
**FACS_blood**	CD44 expressing CD8 T cells; CD8a+CD3e+CD44+	%	2.03 (1.81, 2.49)	1.94 (1.48, 2.42)	2.26 (1.87, 2.79)	2.31 (1.84, 3)
**FACS_blood**		%	0.9 (0.9, 1.1)	1 (0.9, 1.1)	0.7 (0.6, 0.7)	0.7 (0.6, 0.7)
**FACS_blood**	Granulocytes (CD11b+Gr1++; as percentage of living leukocytes)	%	9.84 (7.72, 10.6)	8.09 (7.44, 12.1)	8.67 (7.92, 9.9)	8.3 (6.72, 13.15)

**FACS_blood**	L-selectin expressing CD8 T cells; CD8a+CD3e+CD62L+	%	70.5 (58.1, 78.5)	73.2 (63.85, 78.9)	76.4 (67.4, 79.2)	77 (74.35, 79.95)
**FACS_blood**	Monocytes (gated on non-NK cells, non-Granulocytes, CD11b+ cells; shown as percentage of living leukocytes)	%	7.94 (6.97, 8.57)	7.23 (6.56, 9.03)	7.45 (6.66, 8.61)	6.36 (5.96, 7.14)
**FACS_blood**	CD11b expressing NK cells; CD11b+NK+CD5-	%	73 (70.95, 74.35)	75.5 (72.9, 77.1)	63.3 (60.25, 65.05)	63.8 (57.95, 68.35)
**FACS_blood**	NK cells (pregated on non-T cells; shown as percentage of living leukocytes)	%	3.48 (2.9, 4.23)	3.31 (3.01, 3.74)	2.88 (2.34, 3.11)	3.02 (2.8, 3.38)
**FACS_blood**	NK T cells; NK+CD5+	%	0.64 (0.56, 0.7)	0.64 (0.57, 0.66)	0.53 (0.46, 0.6)	0.5 (0.46, 0.55)
**Grip_strength**	Forelimb grip strength measurement-mean	g	96 (90.5, 101)	97 (91, 102)	135 (130, 142)	110 (104.5, 115)
**Grip_strength**	Forelimb and hindlimb grip strength measurement-mean	g	184 (181.5, 198)	193 (176.5, 201)	221 (205, 228.5)	200 (190, 214)
**Hematology_1**	Hematocrit - percentage of cellular components on whole blood	percent	53.6 (52.2, 56.3)	53.5 (51.4, 54.3)	55.3 (53.6, 56.2)	54.6 (52.8, 55.6)
**Hematology_1**	Hemoglobin concentration in whole blood	g/dl	15.4 (15.1, 16)	15.2 (14.7, 15.5)	15.3 (15.2, 15.8)	15.2 (14.8, 15.5)
**Hematology_1**	Calculated mean corpuscular hemoglobin content of erythrocytes	pg	14.7 (14.5, 15.1)	14.5 (14.4, 14.8)	14.1 (14, 14.3)	14.2 (14, 14.3)
**Hematology_1**	Calculated mean corpuscular hemoglobin concentration of erythrocytes	g/dl	28.9 (28.7, 29.1)	28.7 (28.2, 28.9)	28.1 (27.7, 28.4)	28.2 (27.9, 28.4)
**Hematology_1**	Mean corpuscular volume	fl	51 (50, 52)	51 (51, 52)	50 (50, 51)	50 (50, 51)
**Hematology_1**	Mean platelets volume	fl	5.2 (5.2, 5.2)	5.2 (5.2, 5.3)	5.3 (5.2, 5.4)	5.3 (5.2, 5.4)
**Hematology_1**	Absolute number of eosinophile in whole blood	$10^3/mm^3$	0.1 (0.1, 0.1)	0.1 (0.1, 0.1)	0.1 (0.1, 0.1)	0.1 (0.1, 0.1)
**Hematology_1**	Absolute number of neutrophile granulocytes in whole blood	$10^3/mm^3$	1.5 (1.35, 1.7)	1.45 (1.15, 1.87)	1.9 (1.6, 2.15)	1.7 (1.5, 1.9)
**Hematology_1**	Absolute number of lymphocytes in whole blood	$10^3/mm^3$	4.7 (4.4, 6)	5.4 (4.6, 6.27)	7 (5.9, 7.45)	6.3 (5.2, 7.65)
**Hematology_1**	Absolute number of monocytes in whole blood	$10^3/mm^3$	0.3 (0.3, 0.4)	0.3 (0.3, 0.4)	0.4 (0.3, 0.45)	0.3 (0.25, 0.35)

**Hematology_1**	Percentage of eosinophiles in total white blood cells	%	1.3 (1.1, 1.5)	1.4 (1, 1.7)	1 (0.8, 1.3)	1.1 (1, 1.2)
**Hematology_1**	Percentage of neutrophile granulocytes in total white blood cells	%	19.8 (17.45, 22.95)	18.45 (17.45, 20.6)	18.6 (17.45, 20.4)	17.4 (16.5, 23.7)
**Hematology_1**	Platelet count in whole blood	$10^3/mm^3$	1215 (1134, 1378)	1212 (1180, 1260)	1584 (1500, 1648)	1592 (1422, 1624)
**Hematology_1**	Percentage of lymphocytes in total white blood cells	%	74.8 (71.6, 76.9)	76.15 (74.23, 77.65)	77 (75.15, 78.2)	78.9 (71.4, 79.65)
**Hematology_1**	Percentage of monocytes in total white blood cells	%	5.2 (4.95, 5.9)	5 (4.4, 6.05)	4.4 (4.25, 4.75)	4.1 (3.6, 4.5)
**Hematology_1**	Red blood cell count in whole blood	Mio/mm^3	10.48 (10.23, 10.88)	10.45 (10.11, 10.59)	10.99 (10.72, 11.25)	10.69 (10.48, 11.09)
**Hematology_1**	Red cell distribution width (coefficient of variation)	percent	14 (13.9, 14.2)	14 (13.6, 14.4)	13.7 (13.4, 13.9)	13.6 (13.2, 13.9)
**Hematology_1**	Total white blood cell count in whole blood	$10^3/mm^3$	6.4 (6, 8)	7.4 (6.5, 8.9)	9.7 (8.4, 10.3)	8.3 (6.9, 10.1)
**Hotplate**	time of first response in hotplate test	s	9.7 (6.2, 12.3)	11.1 (10.1, 12.9)	9.6 (7, 12.1)	8.4 (6.7, 11.9)
**Hotplate**	time of 2nd response in hotplate test	s	14.7 (10.4, 20)	16 (13.8, 20.1)	17.5 (15.1, 20.5)	18.2 (14.7, 21.6)
**Immunoglobulins_1**	anti DNA autoantibodies in plasma	OD	0.279 (0.248, 0.353)	0.267 (0.242, 0.288)	0.216 (0.198, 0.243)	0.262 (0.243, 0.306)
**Immunoglobulins_1**	Immunoglobulin A concentration in plasma	µg/ml	693.53 (584.42, 714.56)	631.69 (468.78, 750.89)	710.12 (555.57, 874.5)	513.71 (381.88, 640.53)
**Immunoglobulins_1**	Immunoglobulin G1 concentration in plasma	µg/ml	128.63 (84.31, 145.09)	98.16 (79.56, 134.23)	66.62 (57.28, 93.36)	67.19 (60.25, 85.74)
**Immunoglobulins_1**	Immunoglobulin G2a concentration in plasma	µg/ml	65.56 (51.57, 75.01)	63.96 (55.02, 72.52)	47.02 (43.93, 61.41)	51.07 (41.13, 54.64)
**Immunoglobulins_1**	Immunoglobulin G2b concentration in plasma	µg/ml	441.89 (377.38, 512.73)	399.29 (352.08, 454.11)	222.34 (171.32, 265.65)	250.86 (211.55, 257.51)
**Immunoglobulins_1**	Immunoglobulin G3 concentration in plasma	µg/ml	211.47 (203.83, 280.67)	229.96 (181.57, 317.72)	205.63 (158.62, 246.33)	201.37 (168.47, 234.37)
**Immunoglobulins_1**	Immunoglobulin M concentration in plasma;CD3-CD19+/CD8+CD4-	µg/ml	441.44 (390.33, 515.04)	494.68 (424.4, 537.72)	385.24 (332.95, 527.92)	422.94 (389.17, 521.33)
**Immunoglobulins_1**	Rheumatoid factor in plasma	OD	0.38 (0.32, 0.48)	0.31 (0.26, 0.38)	0.27 (0.22, 0.31)	0.26 (0.24, 0.29)
**Immunoglobulins_2**	Immunoglobulin E concentration in plasma	ng/ml	14.5 (12.5, 28.8)	14.9 (10.2, 21.4)	17 (10.1, 21.7)	13.6 (10.9, 15)
**IPGTT**	Blood glucose concentration at T0	nmol/l	4.6 (4.2, 5.1)	4.5 (4.4, 4.9)	6.3 (5.5, 7.2)	7 (5.6, 7.5)

**IPGTT**	Blood glucose concentration 5 minutes after at T0	nmol/l	18.5 (17.6, 20)	20.1 (18.4, 20.8)	24.3 (23.1, 25.6)	22.1 (20.7, 25.4)
**IPGTT**	Blood glucose concentration 30 minutes after at T0	nmol/l	13.3 (12.3, 14.6)	12.9 (12.4, 15.8)	23.4 (20.8, 26.4)	22.5 (19.7, 24.3)
**IPGTT**	Blood glucose concentration 60 minutes after at T0	nmol/l	8.4 (7.7, 8.8)	8.7 (8.2, 9.2)	13.7 (12.9, 15.6)	13.8 (13, 15.6)
**IPGTT**	Blood glucose concentration 120 minutes after at T0	nmol/l	6.1 (5.3, 6.6)	6.2 (5.5, 6.6)	8.8 (7.3, 9.1)	8.3 (8, 9.3)
**Lung_function**	lung compliance	ml/cm H2O	0.0528 (0.0507, 0.055)	0.0572 (0.0538, 0.0604)	NA (NA, NA)	NA (NA, NA)
**Lung_function**	dynamic compliance of respiratory system (breathing rate 130/min)	ml/cm H2O	0.0226 (0.0221, 0.0236)	0.0237 (0.0222, 0.0252)	NA (NA, NA)	NA (NA, NA)
**Lung_function**	Expiratory reserve volume, FVC - IC	ml	0.29 (0.27, 0.35)	0.29 (0.29, 0.3)	NA (NA, NA)	NA (NA, NA)
**Lung_function**	Volume expired in first 100 ms of fast expiration	ml	0.782 (0.751, 0.837)	0.835 (0.819, 0.889)	NA (NA, NA)	NA (NA, NA)
**Lung_function**	the volume in the lungs at the end-expiratory position	ml	0.248 (0.227, 0.276)	0.248 (0.238, 0.262)	NA (NA, NA)	NA (NA, NA)
**Lung_function**	Forced Vital Capacity, volume expired during fast expiration	ml	0.794 (0.757, 0.853)	0.861 (0.838, 0.905)	NA (NA, NA)	NA (NA, NA)
**Lung_function**	inspiratory Capacity, volume inspired during slow inspiration	ml	0.665 (0.64, 0.694)	0.706 (0.678, 0.749)	NA (NA, NA)	NA (NA, NA)
**Lung_function**	Peak Expiratory Flow, maximum speed of expiration	ml/sec	29.1 (28.7, 30.1)	29.6 (29.5, 29.7)	NA (NA, NA)	NA (NA, NA)
**Lung_function**	Resistance, measurement performed with Buxco FinePointeRC System	cm H2O/ml/sec	1.39 (1.35, 1.42)	1.33 (1.28, 1.39)	NA (NA, NA)	NA (NA, NA)
**Lung_function**	the volume in the lungs at maximal inflation, the sum of VC and RV	ml	0.0446 (0.0446, 0.0446)	0.0221 (0.0217, 0.0225)	NA (NA, NA)	NA (NA, NA)
**Lung_function**	the volume in the lungs at maximal inflation, the sum of VC and RV	ml	0.899 (0.886, 0.948)	0.966 (0.925, 0.997)	NA (NA, NA)	NA (NA, NA)
**Lung_function**	that volume of air moved into or out of the lungs during quiet breathing	ml	0.214 (0.213, 0.216)	0.218 (0.215, 0.22)	NA (NA, NA)	NA (NA, NA)

**Lung_function**	the volume of air breathed out after the deepest inhalation.	ml	0.98 (0.97, 1)	1 (0.96, 1.08)	NA (NA, NA)	NA (NA, NA)
**OCT**	Number of main vessels in the left fundus, observed by optical coherence tomography procedure		10 (9, 11)	10 (9, 11)	10 (9, 11)	10 (9, 11)
**OCT**	Retinal thickness in the left eye, observed by the optical coherence tomography procedure	µm	250 (248, 253)	248 (246, 250)	252 (251, 254)	246 (246, 250)
**OCT**	Number of main vessels in the right fundus, observed by optical coherence tomography procedure		9 (9, 10)	9 (8, 10)	10 (8, 10)	10 (10, 11)
**OCT**	Retinal thickness in the right eye, observed by the optical coherence tomography procedure	µm	254 (247, 259)	250 (246, 256)	249 (247, 252)	247 (244, 252)
**Open_field**	Distance travelled - Total; Distance travelled total	cm	14136.8 (13576.1, 20093.9)	17208 (15358.2, 19252.2)	17001.9 (14144, 20238.6)	17647.6 (16362.5, 19009.8)
**Open_field**	Number of rears - Total		71 (60, 76.5)	86 (70, 91)	89 (69, 103)	80 (73, 104.5)
**Open_field**	% distance in the centre - Total	%	29.2 (26.1, 34)	31.5 (26.2, 32.7)	22.2 (19.7, 26.9)	27.4 (20.6, 31.9)
**Open_field**	% time spent in the centre - Total	percent	18 (16.9, 25.4)	21.4 (15.1, 25.1)	16.4 (13.6, 18)	22.2 (15.8, 27.4)
**Organ_weights**	Heart weight	mg	127 (121, 129)	132 (126, 136)	158 (153, 160)	149 (148, 151)
**Organ_weights**	Liver weight	g	0.997 (0.981, 1.05)	0.975 (0.938, 1.091)	1.29 (1.164, 1.31)	1.351 (1.32, 1.524)
**Organ_weights**	Spleen weight	g	0.092 (0.088, 0.096)	0.098 (0.096, 0.134)	0.072 (0.067, 0.082)	0.08 (0.07, 0.083)
**Organ_weights**	Tibia length	mm	17.6 (17.39, 17.92)	17.35 (17.3, 17.5)	18.24 (18.09, 18.28)	18.1 (17.98, 18.47)
**qNMR**	Fat mass determination at NMR	g	4.8 (4.4, 5.1)	4.6 (4.4, 4.8)	5.8 (5.5, 6.2)	6.2 (5.7, 6.5)
**qNMR**	Lean mass determination at NMR	g	12.9 (12.7, 13.4)	12.6 (12.5, 13.1)	17.3 (16.4, 17.7)	17.4 (16.8, 18.5)
**Rotarod**	Latency to fall-trial 1	s	192 (114, 207.5)	144 (62.5, 193)	126 (58, 149)	117 (89.5, 151)
**Rotarod**	Latency to fall-trial 2	s	211 (168.5, 227)	172 (65, 214)	180 (151, 192.5)	170 (160.5, 189.5)
**Rotarod**	Latency to fall-trial 3	s	200 (170.5, 226)	225 (205, 240)	186 (113, 201.5)	189 (152, 219.5)
**Rotarod**	latency to fall - mean of trials	s	180 (156, 206)	176 (144, 198)	164 (132, 176)	159 (136, 180)
**Scheimpflug**	Mean density value of the left	%	5.5 (5.2, 5.7)	5.5 (5.3, 5.7)	5.6 (5.5, 5.8)	5.7 (5.5, 5.9)

	lens					
**Scheimpflug**	Mean density value of the right lens	%	5.1 (4.8, 5.4)	5.2 (5, 5.4)	5 (4.8, 5.2)	5.3 (5.1, 5.6)
**SHIRPA**	Locomotor Activity	squares crossed/30sec	30 (24, 32)	30 (28, 33)	26 (24, 30)	27 (23, 30)
**Steroids**	Plasma level of corticosterone	nmol/l	412.7 (285.7, 585.9)	577.3 (383.9, 711.5)	136.2 (102.9, 189.8)	110.3 (57.4, 160)
**Virtual_drum**	threshold of spatial frequency	cyc/deg	0.347 (0.34, 0.362)	0.344 (0.33, 0.366)	0.353 (0.343, 0.37)	0.339 (0.328, 0.374)
